# Nurses Role in the Community

**DOI:** 10.5812/nms.11714

**Published:** 2013-06-27

**Authors:** Mohsen Adib-Hajbaghery

**Affiliations:** 1 Department of Medical-Surgical Nursing, Faculty of Nursing and Midwifery, Kashan University of Medical Sciences, Kashan, IR Iran

**Keywords:** Public Health Nursing, Delivery of Health Care, Iran, Delivery of Health Care, Iran

Nurses in the world are active in two main systems: hospital based nursing and community health nursing (CHN). Many countries such as Canada, Ireland, United States of America, Australia and several European countries established the CHN to expand the range and the quality of health care throughout their communities (1, 2) and to realize the justice in the health care system. This specialty of nursing has responsibility to care for healthy and sick persons in all situations and plays key roles in health promotion, health education, disease prevention, emergencies and healthcare services before and after a disaster (3-6). Iran is a large country in Asia with a population of more than 75 million who are aging rapidly and it is predicted that its aged population will reach to 19% by 2030 (7). Although the health indicators of Iran show improvement in the past three decades, its burden of disease shows a shift toward non-communicable diseases such as cardiovascular disorders, diabetes, and high blood pressure. Also road traffic injuries are increasing (8). All these issues indicate the necessity for an advanced health-care system with competent nurses as front-line staff. However, evidences indicate that a disease centered health care system , mostly based on financial profit of a special group, is dominant and negatively affected the performance of other health care professionals such as nurses (7). Along with the suggestion of world health organization for attention to hypertension in 2013, the project of “monitoring the health of Iranian population” with an special focus on screening for high blood pressure and obesity was suggested by Iranian nursing organization and has been operated countrywide by more than 100000 nurses and nurse students in April 2013. This can be the first step for the Iran’s government to pay attention to the strengths and potentials of nurses for monitoring the public health, screening, disease prevention, health education and health promotion. There are lots of evidences that emphases the key role that nurses can play in screening, health monitoring, health promotion, efficacy, and cost effectiveness of their services (9-12). It is for several decades that a major in public health nursing is started but none of the graduates are active in community health care as this position was not defined in our health care system. Recently, the government is starting to restructure the health care system. Although the family physician project is starting, but it is not sufficient and will be expensive for the government and public. Community health nursing may be a complementary project that can help not only the family physician project but can compensate its shortcomings. However, at first step the role of public health nurse should be defined in the structure of the health care system. This can help the government to realize the aim of “Health for All”.
